# P-331. Patient Perspectives Regarding the Use of Contact Precautions for Methicillin-Resistant *Staphylococcus aureus* in a VA Acute Care Hospital

**DOI:** 10.1093/ofid/ofae631.534

**Published:** 2025-01-29

**Authors:** Lyndsay M O’Hara, Mary Bahr-Robertson, Michelle Newman, Lisa Pineles, Michael Rubin, Karim Khader, Richard Nelson, Gio Baracco, Matthew B Goetz, Martin Evans, Kathleen A Linder, Eli N Perencevich, Anthony Harris

**Affiliations:** University of Maryland School of Medicine, Baltimore, Maryland; University of Maryland Baltimore, Baltimore, Maryland; University of Maryland Baltimore, Baltimore, Maryland; University of Maryland School of Medicine, Baltimore, Maryland; University of Utah, Salt Lake City, Utah; University of Utah, Salt Lake City, Utah; University of Utah, Salt Lake City, Utah; Miami VA Healthcare System, Miami, Florida; VA Greater Los Angeles Healthcare System, Los Angeles, California; Veterans Affairs, Lexington, KY; University of Michigan/Ann Arbor VAMC, Ann Arbor, Michigan; University of Iowa/Iowa City VAMC, Iowa City, Iowa; University of Maryland School of Medicine, Baltimore, Maryland

## Abstract

**Background:**

The optimal approach to using contact precautions for patients known to be colonized or infected with MRSA remains unclear despite knowledge that transmission risk varies by healthcare personnel role and care activity being performed. Additionally, the patient perspective is not routinely represented in infection control policies. The aim of this study was to assess patient perspectives on risk-based application of MRSA contact precautions in VA hospitals.

**Figure:**
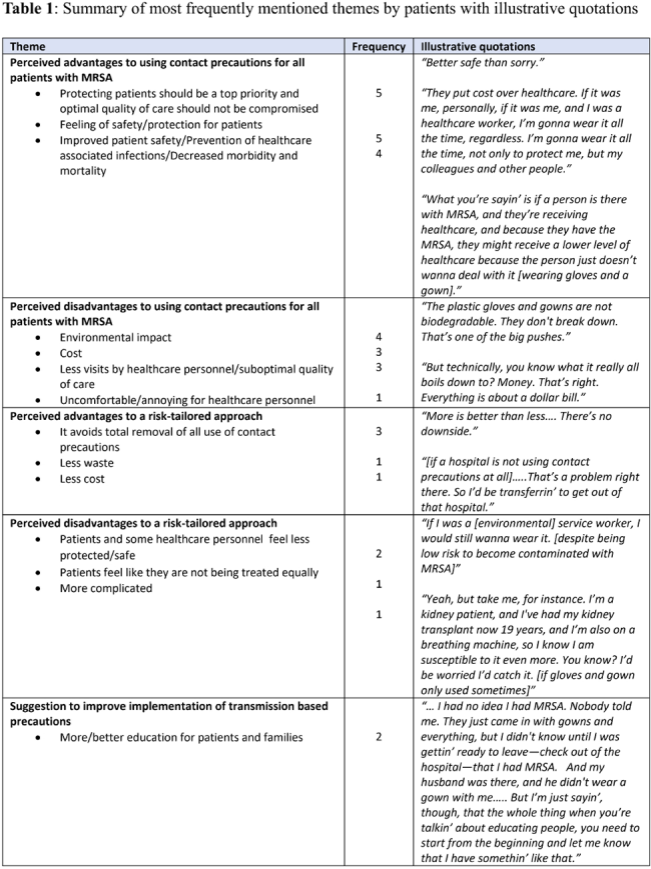

**Methods:**

We conducted an in-person 75-minute focus group with seven Veteran patients colonized with MRSA. The session was audio recorded and transcribed verbatim. We developed a codebook based on the interview guide and used Nvivo 9 for analysis. One research team member coded the transcripts. Thematic analysis was utilized to summarize the findings. Emergent themes and patterns were identified by initial inductive analysis and themes were condensed into overarching categories.

**Results:**

All patients agreed that they would prefer if healthcare workers wore gloves and a gown the entire time when providing care to all patients with MRSA. As shown in Table 1, participants identified three themes related to perceived advantages to the use of contact precautions for all care of patients with MRSA. The most frequently mentioned advantage was that doing so aligns with the premise that protecting patients and making them feel safe while in hospital should be the top priority. Participants also acknowledged five themes describing disadvantages to contact precautions such as the environmental impact and cost but stressed that these disadvantages should not be used to rationalize provision of what they viewed as suboptimal care. Most participants were open to a risk-tailored approach instead of total removal of all contact although some articulated concerns this may make patients may feel less safe and feel like they are not being treated equally.

**Conclusion:**

These qualitative findings suggest that Veteran patients with MRSA support the use of contact precautions and are willing to consider a risk-tailored approach to glove and gown use if this is accompanied by education for healthcare personnel, patients, and families.

**Disclosures:**

**Anthony Harris, MD, MPH**, Innoviva: Advisor/Consultant|UpToDate: Infection Control Editor

